# Elevated Striatal Dopamine Function in Immigrants and Their Children: A Risk Mechanism for Psychosis

**DOI:** 10.1093/schbul/sbw181

**Published:** 2017-01-05

**Authors:** Alice Egerton, Oliver D. Howes, Sylvain Houle, Kwame McKenzie, Lucia R. Valmaggia, Michael R. Bagby, Huai-Hsuan Tseng, Michael A. P. Bloomfield, Miran Kenk, Sagnik Bhattacharyya, Ivonne Suridjan, Chistopher A. Chaddock, Toby T. Winton-Brown, Paul Allen, Pablo Rusjan, Gary Remington, Andreas Meyer-Lindenberg, Philip K. McGuire, Romina Mizrahi

**Affiliations:** 1 Institute of Psychiatry, Psychology and Neuroscience, King’s College London, London, UK;; 2 Research Imaging Center, CAMH, PET Centre, Toronto, ON, Canada;; 3 Department of Psychiatry, Faculty of Medicine, University of Toronto, Toronto, ON, Canada;; 4 Campbell Family Mental Health Research Institute, Centre for Addiction and Mental Health, Toronto, Ontario, Canada;; 5 Department of Psychology, University of Toronto;; 6 Division of Psychiatry, University College London, London, UK;; 7 Psychiatric Imaging Group, MRC Clinical Sciences Centre, Hammersmith Hospital, London, UK;; 8 Department of Psychology, Whitelands College, University of Roehampton, London, UK;; 9 Central Institute of Mental Health, University of Heidelberg/Medical Faculty Mannheim, Mannheim, Germany; 10 These authors are joint first authors.; 11 These authors are joint last authors.

**Keywords:** schizophrenia, stress, positron emission tomography

## Abstract

Migration is a major risk factor for schizophrenia but the neurochemical processes involved are unknown. One candidate mechanism is through elevations in striatal dopamine synthesis and release. The objective of this research was to determine whether striatal dopamine function is elevated in immigrants compared to nonimmigrants and the relationship with psychosis. Two complementary case–control studies of in vivo dopamine function (stress-induced dopamine release and dopamine synthesis capacity) in immigrants compared to nonimmigrants were performed in Canada and the United Kingdom. The Canadian dopamine release study included 25 immigrant and 31 nonmigrant Canadians. These groups included 23 clinical high risk (CHR) subjects, 9 antipsychotic naïve patients with schizophrenia, and 24 healthy volunteers. The UK dopamine synthesis study included 32 immigrants and 44 nonimmigrant British. These groups included 50 CHR subjects and 26 healthy volunteers. Both striatal stress-induced dopamine release and dopamine synthesis capacity were significantly elevated in immigrants compared to nonimmigrants, independent of clinical status. These data provide the first evidence that the effect of migration on the risk of developing psychosis may be mediated by an elevation in brain dopamine function.

## Introduction

Geographic mobility has a large impact on sociological, economic, and health factors within communities. Over 247 million people, or 3.5% of the world population, became migrants over the last decade.^1,2^ One of the most consistent findings in the epidemiology of schizophrenia is the high incidence of the disorder among immigrant groups (relative risk vs nonimmigrant: 2.9).^3^ The risk is particularly increased in immigrant groups who migrate from a country where the population is predominantly black skinned to a country where the population is predominantly white skinned (relative risk vs nonimmigrant: 4.8).^3^ This increased risk of schizophrenia has been reported both in immigrants and in their children. These findings have been replicated in a number of high income countries: The Netherlands,^4^ Denmark,^5^ Germany,^6^ the United Kingdom,^7^ and Canada,^8^ clearly establishing that the incidence of schizophrenia is higher among migrant groups as compared to host populations.

Models of how social factors such as immigration lead to psychosis have mainly considered the role of stress.^9^ Dysregulation of the stress response is a potential etiological factor in the development and relapse of dopamine (DA)-related human disorders including drug-induced psychosis and schizophrenia.^10,11^ The stress-vulnerability model suggests that an endogenous, organic diathesis or vulnerability interacts with internal or external stressors in the development of psychotic disorders.^12^ This includes the social defeat hypothesis, which suggests that social defeat stress may lead to psychosis through sensitization of striatal DA neurotransmission.^13,14^ Initial studies in healthy volunteers have examined the impact of various forms of social defeat on brain function,^15–18^ including striatal DA release.^17,18^ Two recent studies showed that urban living^15^ and immigration^16^ were associated with altered brain responses to stress, providing the first link between social factors and brain function. Particularly in immigrants, perceived discrimination of participant’s ethnic group predicted activation of the ventral striatum,^16^ an area with dense DA innervation. However, so far no study has directly investigated dopamine function in immigrants.

DA hyperactivity in the striatum is a core neurobiological feature of schizophrenia that could underlie the exaggerated incidence of schizophrenia in immigrants.^19–23^ Here, we tested this hypothesis directly using positron emission tomography (PET) in vivo in immigrants and their children with, or at risk for, schizophrenia. To further validate our finding we report two complementary PET DA neuroimaging approaches to study two immigrant populations, one in Canada and one in the United Kingdom. The Canadian study investigated whether the induction of stress by a validated laboratory psychosocial task (Montreal Imaging Stress Task, MIST)^24^ elicits more DA release in immigrants (first and second generation) as compared to the host population. The UK study sought to confirm the association between immigration (first and second generation) and elevated striatal DA function in a different cohort using the complementary technique of [^18^F]DOPA PET to estimate DA synthesis capacity.^25^

## Methods

### Canada Study: Stress-Induced Striatal Dopamine Release

#### Participants.

All subjects provided written, informed consent to participate. Immigration status determined using the self-reported place of birth of the participants, their parents, and grandparents. The immigrant group (*N* = 26) included first (*N* = 9) and second-generation immigrants (*N* = 8) to Canada (The generation information details were not available in 9 subjects, supplementary table S1). The nonimmigrant group (*N* = 31) had been in Canada for at least 3 generations. The immigrant and nonimmigrant groups were comparable for demographics (table 1).

**Table 1.** Demographic Data by Immigration Status for the Stress-Induced DA Release Study, Canada Site

Demographics	Nonimmigrant, *n* = 30	Immigrant, *n* = 26
Age, years; mean (SD)	25.00 (4.86)	23.85 (4.49)
Education, years; mean (SD)	14.00 (2.12)	14.08 (2.53)
Ethnicity 1/2/3/4/5	28/0/0/1/1	6/0/14/4/2
Clinical status		
HV	17	7
CHR	9	14
SCZ	4	5
Gender		
Male	18	14
Female	12	12
Tobacco smoking status		
Nonsmoker	21	23
Smoker	9	3
Cannabis		
Nonuser	14	15
User (current)	16	11
Cocaine		
Nonuser	26	22
User (previous)	4	4
Amphetamine		
Nonuser	28	26
User (previous)	2	0
Ecstasy		
Nonuser	24	22
User (previous)	6	4
Amount injected (MBq)		
Control task	358.16 (38.11)	343.36 (70.67)
Stress task	364.82 (31.08)	367.04 (38.85)
Specific activity (MBq/nmol)		
Control task	43.32 (18.55)	39.22 (18.99)
Stress task	47.82 (18.68)	41.76 (18.12)
Mass injected (µg)		
Control task	2.30 (0.82)	2.38 (0.85)
Stress task	2.12 (0.85)	2.56 (0.81)

*Note*: SD, standard deviation; HV, healthy volunteer; CHR, clinical high risk; SCZ, schizophrenia; Ethnicity (self-reported): 1: White; 2: mixed/multiple ethnic groups; 3: Asian/Asian Canadian; 4: Black/African/Caribbean/Black Canadian; 5: Other. There are no significant group differences.

The immigrant and nonimmigrant groups included subjects at clinical high risk (CHR) for psychosis (*N* = 23), antipsychotic naïve patients with schizophrenia (SCZ, *N* = 9) and healthy volunteers (HV, *N* = 24) (supplementary table S1). The CHR subjects met criteria for prodromal syndromes^26^ based on the Structured Interview of Prodromal Syndromes (SIPS)^26,27^ and were recruited from the early identification and treatment services for those who are at risk of psychosis (Focus on Youth Psychosis Prevention clinic, FYPP) at the Centre for Addiction and Mental Health (CAMH), Toronto, Canada. SCZ subjects were recruited from the first episode psychosis clinic at CAMH, with the diagnosis of schizophrenia and schizophreniform disorder, no other current Axis I psychotic disorders, and no antipsychotic exposure (ie, antipsychotic naïve). HV were recruited from the same geographic area, had no personal history of psychiatric symptoms, and were not taking psychotropic medication. Participants from the current study overlapped with the cohort in previous dopamine imaging studies^23,28,29^ in whom information on immigration status was available. Exclusion criteria for all participants included pregnancy, other contra-indications to PET imaging and illicit drug use other than cannabis in the 6 months prior to imaging. The absence of illicit substance use other than cannabis was confirmed using urine drug screens at the time of PET imaging. In all participants, the level of perceived stress was measured using the Trier Inventory for the Assessment of Chronic Stress.^30^

#### Montreal Imaging Stress Task.

A psychological stress task was performed during the PET imaging session to elicit dopamine release. Psychological stress was induced using the Montreal Imaging Stress Task (MIST) which has been validated in previous fMRI and PET studies^15,16,23,24,28,31,32^ (see supplementary e-material MIST section for details).

In all experiments, the control or stress task was started ~6–8 min before tracer injection, with 6 min of mathematical questions and ~1–2 min for either neutral or negative verbal feedback. Perception of stress during the control and stress conditions was assessed by a shortened version of the state anxiety questionnaire.^23,28,33^ The stress task was effective in producing subject-tailored failure and eliciting a stress response, which was equivalent across the immigrant and nonimmigrant groups (supplementary figures S1 and S2).

#### [^11^C]-(+)-PHNO PET Image Acquisition.

All 56 subjects completed 2 PET scans (*n* = 112 PET scans) at least a week apart at the same time of the day, one while performing the SMCT (sensory motor control task) and one while undergoing the MIST (stress task). Stress-induced DA release was measured using [^11^C]-(+)-PHNO positron emission tomography (PET), through quantification of the competition between endogenous DA and [^11^C]-(+)-PHNO for D2/3 receptor binding in the striatum. The radiosynthesis of [^11^C]-(+)-PHNO has been described in detail elsewhere.^34^ Studies were carried out using a high resolution PET CT, Siemens-Biograph HiRez XVI (Siemens Molecular Imaging ). Each subject was administered ~333–370MBq of high specific activity [^11^C]-(+)-PHNO and scanned for 90 min. A custom-fitted thermoplastic mask was made for each subject and used with a head fixation system during PET acquisition to minimize head movement. A CT transmission scan was acquired after each emission scan for attenuation correction.

#### [^11^C]-(+)-PHNO PET Image Analysis.

PET images were reconstructed with a 2D filtered back projection algorithm with a ramp filter at Nyquist cut-off frequency and rebinned into 31 time frames (comprising the background frame, followed by [^11^C]-(+)-PHNO injection by fifteen 60-s frames and fifteen 300-s frames) as previously validated.^35^ Time activity curves (TAC) from the regions of interest (ROIs) were obtained from the dynamic [^11^C]-(+)-PHNO PET images. The ROIs selected were the whole striatum and its functional subdivisions, including the associative (AST), limbic (LST), and sensorimotor striatum (SMST).^36^ ROIs were delineated using an automated method implemented in an in-house software (ROMI).^37^ Activity from bilateral ROI were combined and the volume-weighted average signal was used to derive [^11^C]-(+)-PHNO binding potential (BP_ND_).^38^ BP_ND_ was calculated by the Simplified Reference Tissue Method^39^ using a cerebellar reference region, as previously described for [^11^C]-(+)-PHNO.^40^

Stress-induced DA release was quantified as [^11^C]-(+)-PHNO % displacement = (BP_ND_SMCT − BP_ND_ MIST) / BP_ND_ SMCT) × 100%.

In complement to the ROI approach, stress-induced DA release was also assessed using voxel-wise analysis. Each BP_ND_ parametric map was spatially normalized to the Montreal Neurological Institute (MNI) anatomical template using SPM2 normalization and co-registration tools. These maps were then used to assess significant contrasts between nonstress and stress conditions at the level of the whole brain using an implicit mask of BP_ND_ >0.3. This mask restricts the statistical search to areas of specific binding (ie, excluding cerebrospinal fluid, background, and the reference region).

### UK Study: Striatal Dopamine Synthesis Capacity

#### Participants.

All subjects provided written, informed consent to participate. As in the Canada study, immigration status determined using the self-reported place of birth of the participants, their parents, and grandparents. The immigrant group (*N* = 31) included first (*N* = 13) and second-generation (*N* = 18) immigrants to the United Kingdom. The nonimmigrant group (*N* = 44) had been in the United Kingdom for at least 3 generations. The immigrant and nonimmigrant groups were comparable for demographics (table 2).

**Table 2.** Demographic Data by Immigration Status for the DA Synthesis Study, UK Site

Demographics	Nonimmigrant, *n* = 44	Immigrant, *n* = 32
Age, years; mean (SD)	24.61 (4.54)	23.25 (4.14)
Ethnicity 1/2/3/4/5	35/2/0/7/0	9/2/5/16/0
Clinical status		
HV	13	13
CHR	31	19
Gender		
Male	29	15
Female	15	17
Tobacco smoking status		
Nonsmoker	24	20
Smoker	19	13
Cannabis		
0/1/2/3/4	12/14/5/6/7	10/11/4/4/3
Cocaine		
0/1/2/3/4	26/12/3/2/1	23/5/1/2/1
Amphetamine		
0/1/2/3/4	30/12/1/1/0	28/3/0/1/0
Ecstasy		
0/1/2/3/4	21/18/3/2/0	24/4/3/1/0
Injected dose (MBq)	166.41 (16.20)	163.97 (18.21)
Specific activity (MBq/μM)	26.09 (13.94)	28.28 (16.70)

*Note:* SD, standard deviation; HV, healthy volunteer; CHR, clinical high risk; SCZ, schizophrenia; Ethnicity (self-reported): 1L White; 2L Mixed/multiple ethnic groups; 3: Asian/Asian Canadian; 4: Black/African/Caribbean/Black Canadian; 5: Other. Drug use is denoted 0: never used; 1: very occasional or experimental use; 2: occasional (monthly) use; 3: moderate (weekly) use; 4: severe (daily) use. There are no significant group differences.

These participants included 50 individuals who met operationalized CHR criteria^41^ and 26 HV (supplementary table S2). CHR participants were recruited from Outreach and Support in South London (OASIS, part of the South London and Maudsley National Health Service Trust). Of the 50 CHR subjects, 2 were using antipsychotics (1 using olanzapine and 1 using quetiapine). The HV group was recruited from the same geographic area and had no personal of psychiatric symptoms, or psychotropic medication. Both the CHR and HV groups included subjects who had participated in previous dopamine imaging studies^19,21^ where information on immigration status was available. Exclusion criteria for all participants included pregnancy or other contra-indications to PET imaging. The absence of illicit substance use other than cannabis was confirmed by urine drugs screen at the time of PET imaging.

#### [^18^F]-DOPA PET Image Acquisition.

Studies were carried out using either a CTI/Siemens ECAT HR+ 962 tomograph (16 HV; 26 CHR)^19^ or a CTI/Siemens ECAT HR++ 966 tomograph (10 HV; 24 CHR)^21^ (Siemens Molecular Imaging). All participants were asked to fast for 12 h before imaging and received carbidopa (150 mg) and entacapone (400 mg) orally 1 h before imaging to reduce the formation of radiolabeled [^18^F]-DOPA metabolites. Data were acquired in three-dimensional mode while participants lay at rest. Transmission scans performed before radiotracer injection corrected for attenuation and scatter. Head position was minimized using a light head strap. [^18^F]-DOPA was administered by bolus intravenous injection (supplementary table S2). Emission data were acquired in list mode for 95 min, and rebinned into 26 time frames (comprising the 30-s background frame, followed by [^18^F]-DOPA injection with four 60-s frames, three 120-s frames, three 180-s frames, and fifteen 300-s frames).

#### [^18^F]-DOPA PET Image Analysis.

Data were reconstructed using the 3D reprojection algorithms. To correct for head movement during the scan, nonattenuation-corrected dynamic images were denoised using a level 2, order 64 Battle-Lemarie wavelet filter^42^ and individual frames were realigned using a mutual information algorithm.^43^ The transformation parameters were then applied to the corresponding attenuation-corrected frames, and the realigned frames were combined to create a movement-corrected dynamic image for analysis.

Striatal ROI were delineated bilaterally on a single subject T1 MRI in MNI space. The cerebellar reference region was defined using a probabilistic atlas.^44^ An [^18^F]-DOPA template, also in MNI space, was then normalized together with the ROI map to each individual PET summation image using SPM5.^25^ Graphical analysis, adapted for a reference tissue input function^45,46^ was used to estimate presynaptic dopamine synthesis capacity by calculating the rate of utilization of the dopamine precursor ^18^F-DOPA (*k*_i_^cer^ min^−1^) in the bilateral striatum. To control for potential effects of scanner model,^19^ individual subject *k*_i_^cer^ values were converted to *z*-scores [*z* = (*k*_i_^cer^ – scanner mean *k*_i_^cer^) / scanner standard deviation (SD)] for all analyses.

### Statistical Analysis

Analysis of covariance was used to determine the main effects of immigration status and of clinical group on [^11^C]-(+)-PHNO displacement or ^18^F-DOPA *z*-score in the striatum. Nonsignificant immigration × clinical group interaction terms were subsequently removed from the model. Effect sizes were calculated as partial eta^2^. Follow-up analysis co-varied for potential effects of illicit drug use. The voxel-wise analysis of DA release utilized SPM8 to examine the difference between the control and stress conditions using paired *t*-tests in the immigrant and nonimmigrant groups, with cannabis use and clinical status as covariates. Partial correlation examined the relationship between perceived stress and [^11^C]-(+)-PHNO displacement, co-varying for clinical status (*n* = 54). Statistical significance was defined as *P* < .05 unless otherwise stated.

## Results

### Canada Study: Stress-Induced Striatal Dopamine Release

**Fig. 1.**
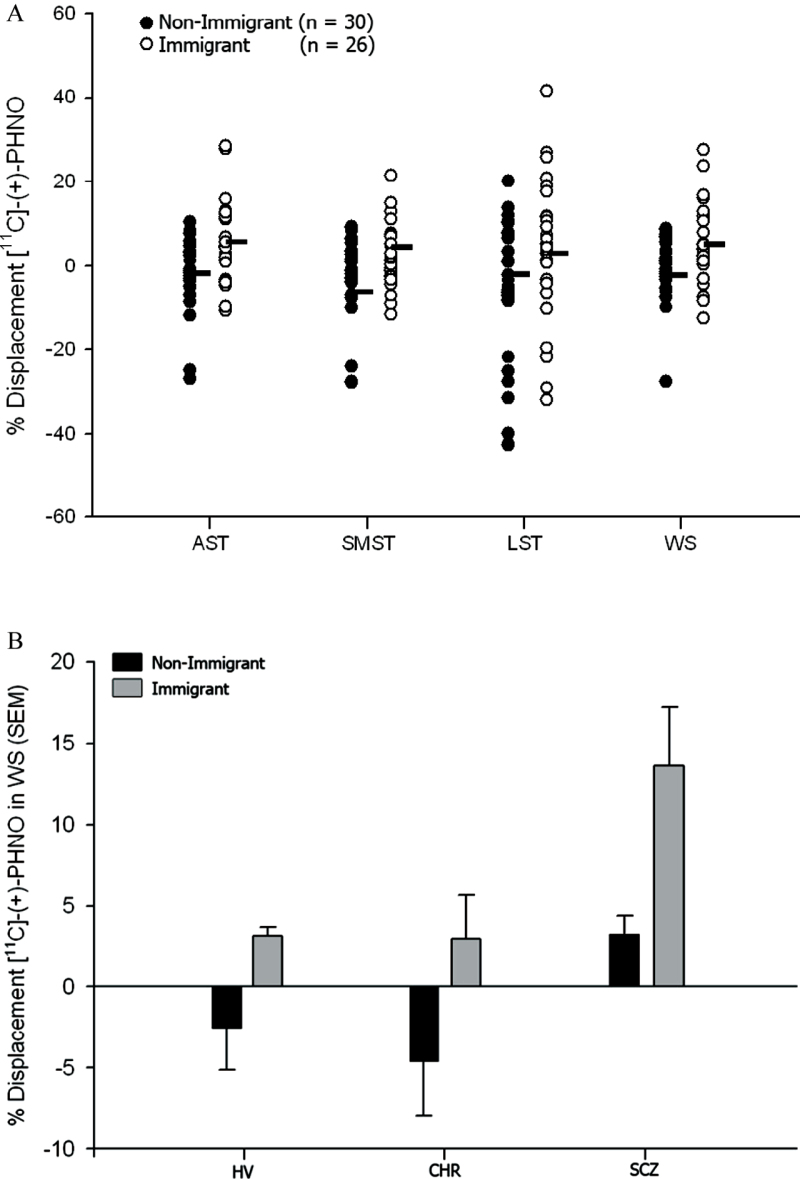
(A) Significant effect of immigration on stress induced DA release in the whole striatum and in striatal subdivisions, including AST, LST, and SMST. WS (*F* = 8.08; *df* = 1, 52; *P* = .006), AST (*F* = 8.05; *df* = 1, 52; *P* = .006), LST (*F* = 5.22; *df* = 1, 52; *P* = .03), and SMST (*F* = 4.17; *df* = 1, 52; *P* = .05). (B) Effects of immigration on stress-induced dopamine release in the whole striatum by clinical vulnerability (mean and standard error of the mean, SEM).

[^11^C]-(+)-PHNO BP_ND_ values in the control and stress conditions are provided in the supplementary table S3. Immigrants demonstrated elevated striatal DA release in response to stress compared to nonmigrant Canadians (*F* = 8.08; *df* = 1, 52; *P* = .006; partial eta2 = 0.13) (figure 1). The clinical group effect was significant (*F* = 3.57; *df* = 1, 52; *P* = .04; partial eta^2^ = 0.12), as patients with schizophrenia showed higher striatal DA release than CHR and HV (Bonferroni corrected comparisons, *P* = .05 and .02, respectively). However, the clinical group by immigration status interaction term was nonsignificant. The significant increase in stress-induced DA release in immigrants was present in the AST (*F* = 8.05; *df* = 1, 52; *P* = .006; partial eta^2^ = 0.13; figure 1), LST (*F* = 5.22; *df* = 1, 52; *P* = .03; partial eta^2^ = 0.09), and SMST (*F* = 4.17; *df* = 1, 52; *P* = .05; partial eta2 = 0.07). Voxel-wise analysis also revealed significant stress-induced striatal DA release in immigrants (supplementary figure S3) with no significant effect in nonimmigrants.

Secondary exploratory ANOVA comparing nonimmigrants to first and second generation immigrants separately found an overall significant effect of immigration on striatal DA release (*F* = 3.31; *df* = 2, 44; *P* = .046), with post hoc tests indicating this was primarily driven by elevated striatal DA release in the first generation immigrants compared to the nonimmigrant group (Bonferroni corrected comparisons, *P* = .05), whereas stress induced DA release did not differ significantly between second generation immigrants and nonimmigrants (*P* = .68) or first generation immigrant groups (*P =* 1.00).

When the ROI analysis was repeated with cannabis use included as a covariate, the effects of both immigration (*F* = 7.26; *df* = 1, 51; *P* = .01) and clinical group (*F* = 3.68; *df* = 2, 51; *P* = .03) remained significant across the whole striatum. The immigration effect remained significant in AST (*F* = 7.23; *df* = 1, 51; *P* = .01) and LST (*F* = 4.48; *df* = 1, 51; *P* = .04), and trend level in SMST (*F* = 3.51; *df* = 1, 51; *P* = .07). The clinical group effect was also significant in AST (*F* = 5.15; *df* = 1, 51; *P* = .01), and was present at trend-level in SMST (*F* = 2.81; *df* = 2, 51; *P* = .07), but not in LST. The effects of immigration and clinical group on DA release also remained after co-varying for lifetime history of previous use of other illicit drugs (table 1). Stress-induced striatal DA release was associated with several measures of social stress, including work overload, social overload, social pressure, social isolation, etc. (supplementary table S1 and supplementary figure S4).

### UK Study: Striatal Dopamine Synthesis Capacity

**Fig. 2.**
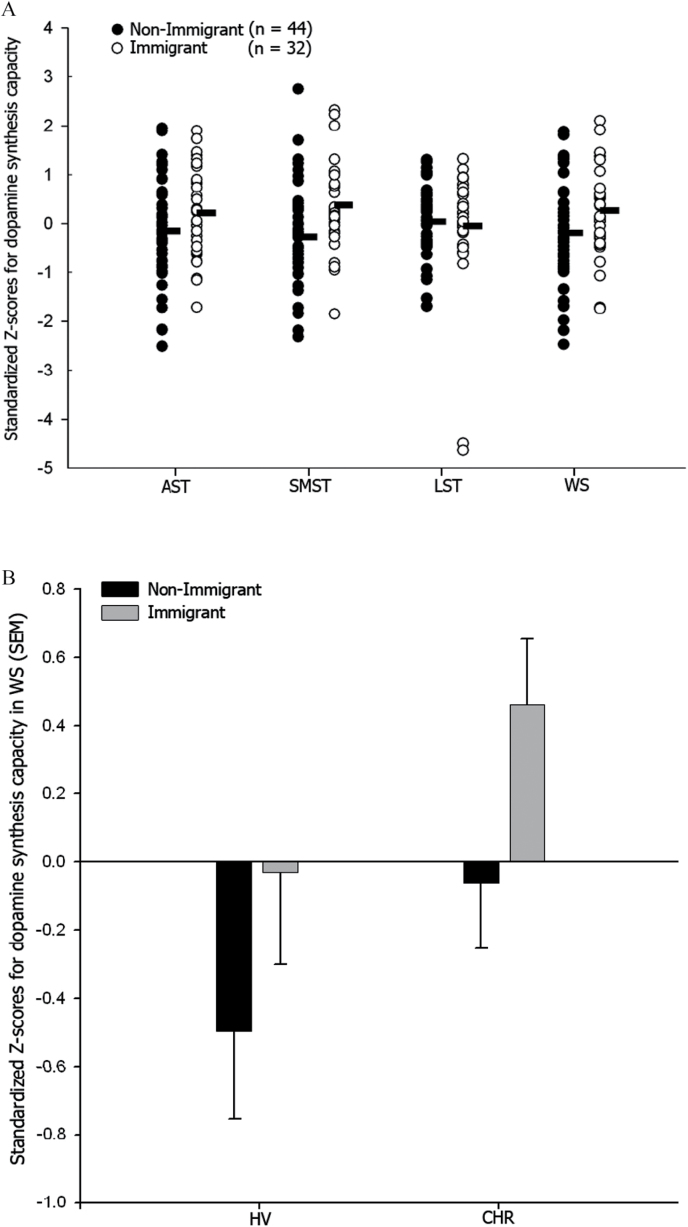
(A) Significant effect of immigration on DA synthesis capacity in the whole striatum and SMST, with similar trends in the AST. WS (*F* = 4.95; *df* = 1, 73; *P* = .03), SMST (*F* = 9.24; *df* = 1, 73; *P* = .003), and AST (*F* = 3.28; *df* = 1, 73; *P* = .07). (B) Effects of immigration on DA synthesis capacity in the whole striatum by clinical vulnerability (mean and standard error of the mean, SEM).

Striatal DA synthesis capacity was elevated in the immigrant compared to the nonimmigrant group (*F* = 4.95; *df* = 1, 73; *P* = .03, partial eta2 = 0.06, figure 2). The clinical group effect showed a trend for increased DA synthesis in the CHR compared to HV group (*F* = 3.79; *df* = 1, 73; *P* = .06; partial eta2 = 0.05). The clinical group by immigration status interaction term was nonsignificant. The significant elevation in DA synthesis capacity in immigrants was present in the SMST (*F* = 9.24; *df* = 1, 73; *P* = .003; partial eta2 = 0.11), with a similar trend in the AST (*F* = 3.28; *df* = 1, 73; *P* = .07; partial eta2 = 0.04). Secondary exploratory ANOVA comparing nonimmigrants to first and second generation immigrants separately found an overall significant effect of immigration on striatal dopamine synthesis capacity (*F* = 3.12; *df* = 1, 73; *P* = .047), with post hoc tests indicating this was primarily driven by elevated DA synthesis capacity in the second generation immigrant compared to nonimmigrant group (*P* = .04), whereas DA synthesis capacity did not differ significantly between first generation immigrant and nonimmigrant (*P* = .91) or second generation immigrant groups (*P =* .23).

When cannabis use was included in the model, the main effects of both immigration and clinical status on DA synthesis capacity were significant for the whole striatum (*F* = 4.83; *df* = 1, 72; *P* = .03 and *F* = 4.35; *df* = 1, 72; *P* = .04, respectively). The immigration effect remained significant in SMST (*F* = 9.06; *df* = 1, 72; *P* = .004) and trend level in AST (*F* = 3.18; *df* = 1, 72; *P* = .07). The clinical group effect was significant in AST (*F* = 4.56; *df* = 1, 72; *P* = .04). The main effects of immigration and clinical group on DA synthesis also remained after co-varying for previous use of other illicit substances (table 2), or after excluding the 2 CHR subjects who were currently taking antipsychotics.

## Discussion

These results indicate that striatal DA function is elevated in both immigrants and their children, including those at risk for psychosis or with schizophrenia, confirming its relevance for psychotic disorders. The elevation in DA in immigrants was present with relatively large effect size in both the Canada and UK studies, which were performed in independent samples using 2 complementary approaches to imaging presynaptic DA function. As in previous studies of schizophrenia,^21^ elevated DA in immigrants were most evident in dorsal striatal regions (AST and SMST). This suggests that adverse psychological, social, and environmental experiences associated with immigration may increase the risk of schizophrenia by influencing brain dopamine function, a key pathophysiological component of psychosis.

The increased DA function in immigrants and their children did not appear to be influenced by cannabis exposure.^28,47^ In both experiments, we confirmed the absence of illicit drugs other than cannabis at the time of PET imaging by urine drugs screen. There were no significant differences in self-reported drug use and immigration effects remained significant when previous illicit drug use was included in the analyses. While lack of detailed illicit drug histories or hair analyses is a limitation, from the available data we believe that our findings were unlikely to be related to drug use. As both the UK and Canadian cohorts included participants of multiple ethnic groups and different generation (1st vs 2nd) distribution, we were unable to determine the effects of immigration accounting for ethnicity or generation status. Future studies could focus on the impact of ethnicity and immigration generation on dopamine function.

While the reasons for the increased risk of schizophrenia and other psychoses in migrant groups are still unclear, there is increasing evidence that victimization, discrimination, social isolation, social defeat, and growing up in an urban environment may contribute.^17,48,49,50^ Our subjects came from 2 large cities (London, UK and Toronto, Canada) with relatively large immigrant populations. One avenue for future research is the extent by which social support interventions can mitigate the relationship between risk factors such as immigration, neurobiological markers, and poor mental health. Given the significant waves of immigration happening at this time both in Europe and the Americas, these interventions may become key as pre-emptive strategies.

Cross-sectional studies such as ours cannot provide causal information about the associations between immigration, elevated dopamine function, and schizophrenia. However, studies in experimental animals have shown that experiences of social defeat stress can lead to striatal DA elevation.^51^ More broadly, our data are consistent with hypotheses linking social defeat to elevated DA and schizophrenia,^13,14,52,53^ and reports of striatal DA elevations in adults with hearing impairment,^17^ history of childhood abuse^18^ or low parental care,^24^ which may all be forms of social defeat/stress. Our study further supports the relevance of the interaction between adverse social factors and striatal DA for psychotic disorders.

## Conclusions

The data from the present study identify a plausible biological mechanism that links the effects of migrant status to the risk of developing psychosis through elevated brain dopaminergic function. These findings suggest that interventions designed to reduce the psychosocial impact of being a migrant might be useful as a means of reducing the risk of psychotic illness.

## Supplementary Material

Supplementary data are available at *Schizophrenia Bulletin* online.

## Funding

The Canadian study was funded by the Canadian Institute of Health Research (CIHR) and the National Association for Research on Schizophrenia and Depression (NARSAD). The UK study was funded by the Medical Research Council (MRC), grant number G0700995, and presents independent research supported by the National Institute of Health Research (NIHR), Biomedical Research Centre at South London, Maudsley NHS Foundation Trust and King’s College London. The views expressed are those of the author(s) and not necessarily those of the NHS, the NIHR or the department of Health.

## Supplementary Material

Supplementary_Material
