# Supplementary material for: Elevated Striatal Dopamine Function in Immigrants and Their Children: A Risk Mechanism for Psychosis
Source: Schizophr Bull. 2017 Jan 5;43(2):293–301. doi: 10.1093/schbul/sbw181 (PMC5605255; doi:10.1093/schbul/sbw181)
Supplement: Supplementary_Material [file sbw181_suppl_Supplementary_Material.doc]

**Figure S1.** **Validation of stress paradigm, DA release study: Comparison of subjective feelings by state anxiety questionnaire after control (SMCT) and stress (MIST) sessions across all groups (n=52).**

All measures are significantly different between two sessions (paired *t*: 5.66~9.85 in positive feeling measures and -7.54 ~ -11.49 in negative feeling measures; all *p* values <0.001 in all measures).


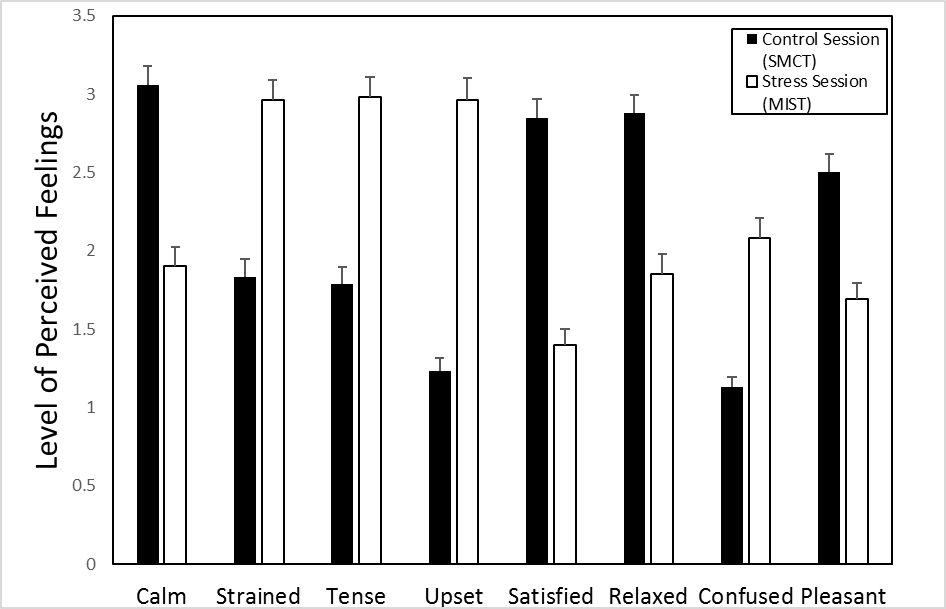


| **Perceived feeling after task** | **SMCT** | **MIST** | **Paired *t*** | ***p*** |
| --- | --- | --- | --- | --- |
| Calm | 3.06(0.87) | 1.90(0.89) | 8.34 | <0.001 |
| Strained | 1.83(0.86) | 2.96(0.95) | -7.54 | <0.001 |
| Tense | 1.79(0.75) | 2.98(0.92) | -9.05 | <0.001 |
| Upset | 1.23(0.61) | 2.96(1.01) | -11.49 | <0.001 |
| Satisfied | 2.85(0.85) | 1.4(0.75) | 9.85 | <0.001 |
| Relaxed | 2.88(0.83) | 1.85(0.92) | 6.91 | <0.001 |
| Confused | 1.13(0.44) | 2.08(0.93) | -8.00 | <0.001 |
| Pleasant | 2.5(0.85) | 1.69(0.76) | 5.66 | <0.001 |

**Figure S2. Comparison of subjective feelings by state anxiety questionnaire after control (SMCT) and stress (MIST) sessions, showing similar effects in both non-immigrant and immigrant.**

For the total scores, the immigration effect was non-significant *(F*=2.218*, p*=0.14*)*, the session effect was significant *(F*=119.114*, p*<0.001*)*, and the immigration*session interaction was non-significant *(F*=1.001*, p=*0.32*)*


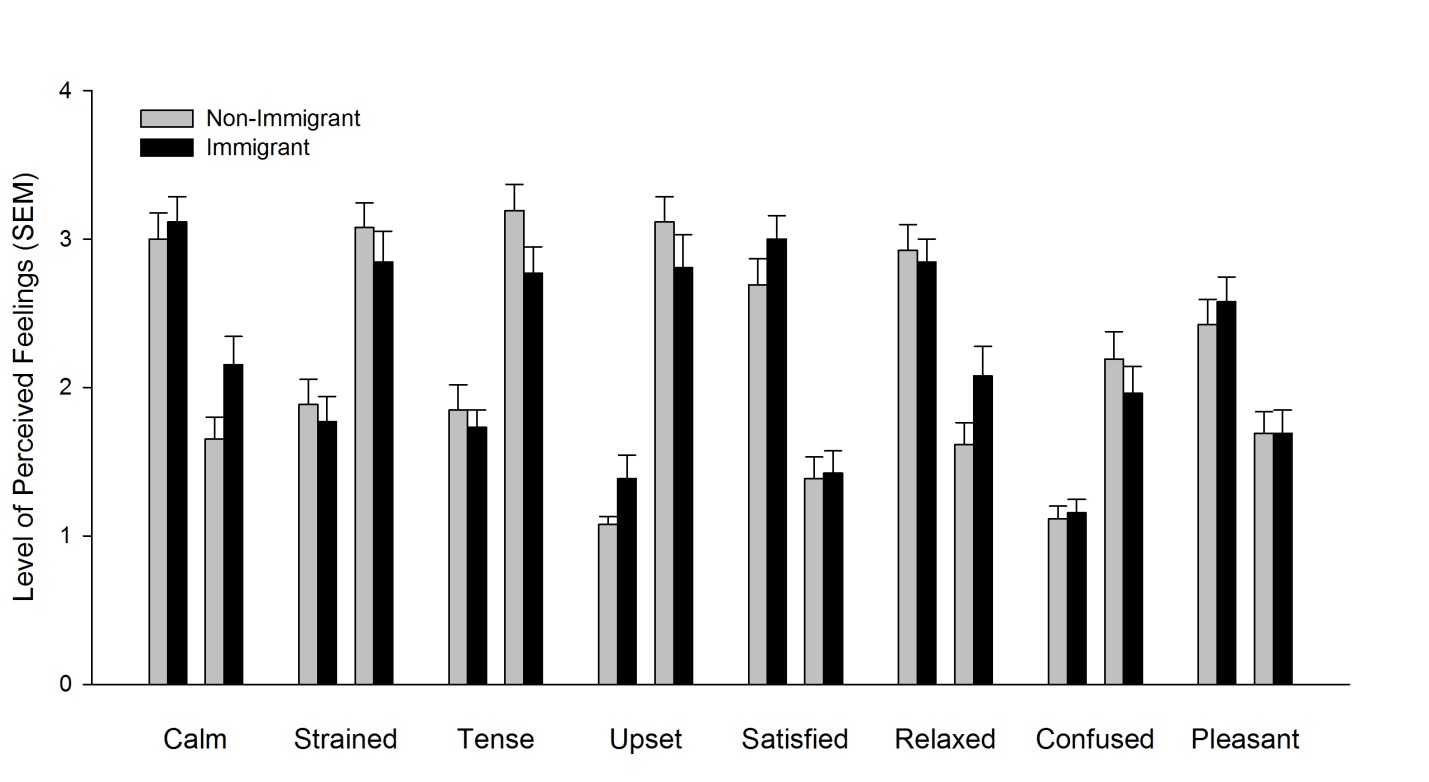


SMST

MIST

SMST

MIST

SMST

MIST

SMST

MIST

SMST

MIST

SMST

MIST

SMST

MIST

SMST

MIST

**Figure S3.** **t-statistical parametric map overlaid on an average T1 MRI**

t-statistical parametric map overlaid on an average T1 MRI, illustrating clusters of significant decrease in [^11^C]-(+)-PHNO binding (BP_ND_) in response to the stress according to immigration status, controlled for cannabis use and group allocation (Left dorsal striatum, AST and SMST, cluster size = 223; *p*<0.006, FWE corrected at the cluster level; peak MNI coordinates: -18, 24, 6, left caudate, *t*_max_=3.94; -16, 20, 6, left putamen; *t*_max_=3.36, with no significant difference between conditions observed in non-immigrants). These significant [^11^C]-(+)-PHNO changes in striatal areas of immigrants suggest increased stress-induced DA release in immigrants as compared to host Canadians.

**
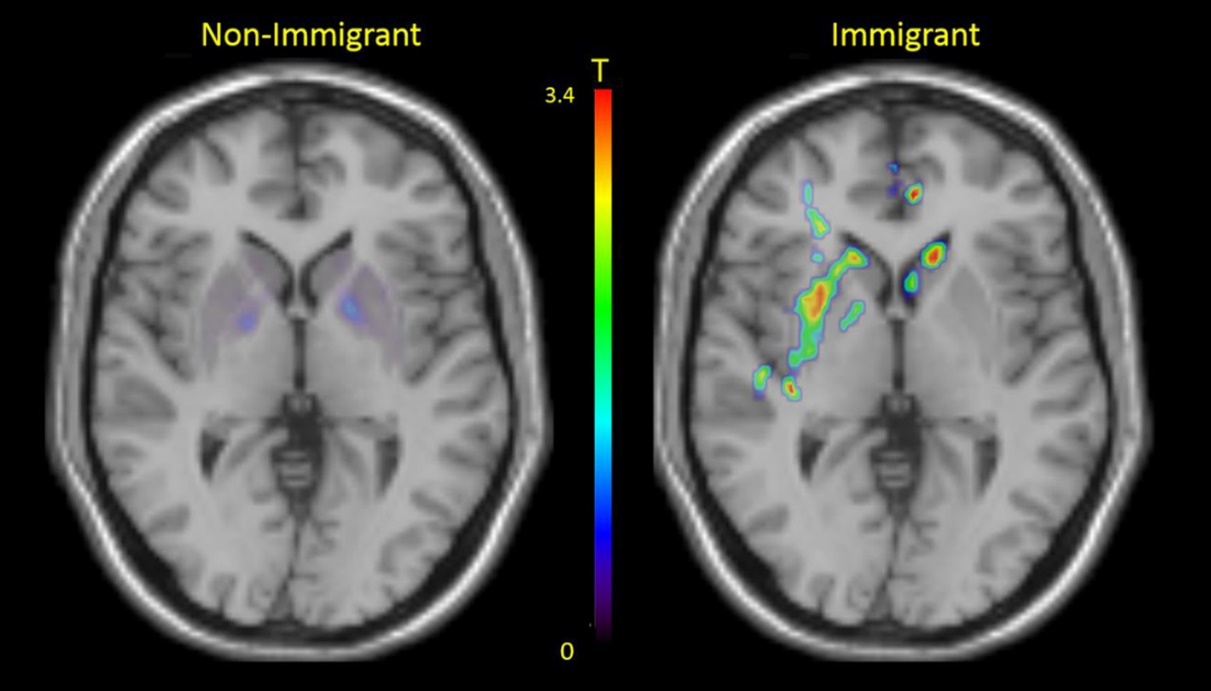
**

**Figure S4. Post-hoc associations of whole striatum (WS) displacement**

**
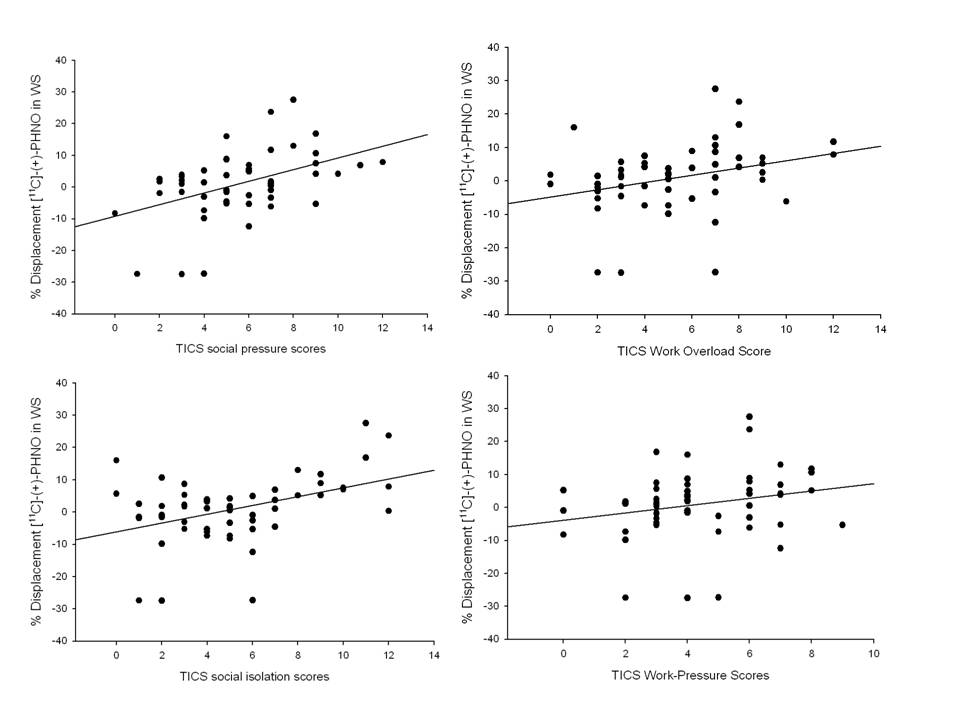
**

Representative figure depicting post-hoc associations whole striatum (WS) displacement, showing significant correlation between [^11^C]-(+)-PHNO displacement in the WS with TICS work overload scores (*r*=0.305, *p*=0.030), work pressure scores (*r*=0.323, *p*=0.021), social pressure scores (*r*=0.360, *p*=0.010) and social isolation scores (*r*=0.381, *p*=0.006).

**Table S1. Demographic variables by clinical status for the stress-induced DA release study, Canada site.**

|  | | **HV n= 24** | **CHR n= 23** | **SCZ n= 9** |
| --- | --- | --- | --- | --- |
| Age, years; mean (SD) | | 25.33 (4.41) | 23.70 (4.76) | 24.11 (5.33) |
| Education, years; mean (SD) | | 14.54 (2.02) | 13.65 (2.60) | 13.63 (2.07) |
| Ethnicity 1/2/3/4/5 | | 17/0/4/2/1 | 12/0/7/3/1 | 5/0/3/0/1 |
| Immigration status | Non-immigrant | 17 | 9 | 4 |
|  | Immigrant (1^st^ /2^nd^ generation/ not available) | 7 (3/1/3) | 14 (2/6/6) | 5 (4/1/0) |
| Gender | Male | 14 | 12 | 6 |
|  | Female | 10 | 11 | 3 |
| Tobacco smoking status | Non-smoker | 20 | 18 | 6 |
|  | Smoker | 4 | 5 | 3 |
| Cannabis | Non-user | 12 | 12 | 5 |
|  | User (current) | 12 | 11 | 4 |
| Cocaine | Non-user  User (previous) | 22  2 | 17  6 | 9  0 |
| Amphetamine | Non-user  User (previous) | 24  0 | 21  2 | 9  0 |
| Ecstasy | Non-user  User (previous) | 20  4 | 17  6 | 9  0 |
| Amount Injected (MBq) | Control Task  Stress Task | 350.02 (51.06)  367.78 (27.38) | 358.53 (61.79)  365.56 (31.82) | 337.44 (54.39)  361.12 (56.98) |
| Specific Activity (mBq/nmol) | Control Task  Stress Task | 42.87 (18.44) 46.32 (16.57) | 41.41 (18.94)  46.59 (22.36) | 37.56 (20.37)  37.45 (10.77) |
| Mass Injected (µg) | Control Task  Stress Task | 2.17 (0.68)  2.09 (0.68) | 2.43 (0.95)  2.43 (1.04) | 2.54 (0.87)  2.65 (0.67) |

SD: Standard deviation; HV: healthy volunteer, CHR: clinical high risk; SCZ: schizophrenia; Ethnicity (self-reported): 1: White; 2: Mixed / Multiple ethnic groups; 3: Asian / Asian Canadian 4: Black / African / Caribbean / Black Canadian; 5: Other.

**Table S2.** **Demographic variables by clinical status for the DA synthesis study, UK site**

|  | | **HV n= 26** | **CHR n= 50** |
| --- | --- | --- | --- |
|  |  |  |  |
| Age, years; mean (SD) | | 23.58 (3.99) | 24.28 (4.62) |
| Ethnicity 1/2/3/4/5 | | 12/2/2/10/0 | 32/2/3/13/0 |
| Immigration status | Non-immigrant  Immigrant (1^st^ /2^nd^ generation) | 13  13 (6/7) | 31  19 (6/13) |
| Gender | Male | 15 | 15 |
|  | Female | 29 | 21 |
| Tobacco smoking status | Non-smoker | 18 | 25 |
|  | Smoker | 8 | 25 |
| Cannabis | 0/1/2/3/4 | 10/9/3/1/3 | 12/16/6/9/7 |
| Cocaine | 0/1/2/3/4 | 24/4/0/1/0 | 28/13/4/3/2 |
| Amphetamine | 0/1/2/3/4 | 22/4/0/0/0 | 36/11/1/2/0 |
| Ecstasy | 0/1/2/3/4 | 18/5/3/0/0 | 27/17/3/3/0 |
| Injected dose (MBq) | | 167.62 (17.38) | 164.21 (16.86) |
| Specific Activity (MBq/μM) | | 26.88 (11.10) | 27.08 (16.92) |

SD: Standard deviation; HV: healthy volunteer, CHR: clinical high risk; SCZ: schizophrenia; Ethnicity (self-reported): 1: White; 2: Mixed / Multiple ethnic groups; 3: Asian / Asian Canadian 4: Black / African / Caribbean / Black Canadian; 5: Other. Drug use is denoted 0: never used; 1: very occasional or experimental use; 2: occasional (monthly) use; 3: moderate (weekly) use; 4: severe (daily) use. There are no significant group differences.

**Table S3. [^11^C]-(+)-PHNO binding potential (BP_ND_) in the control and stress conditions in Immigrants versus Non-Immigrants**

|  | **Non-immigrant (n=30)** | | | | **Immigrant (n=26)** | | | |
| --- | --- | --- | --- | --- | --- | --- | --- | --- |
| **Region** | **BP_ND_ Control Task** | **BP_ND_ Stress Task** | **% Displacement** | **Paired T-test (df=29)** | **BP_ND_ Control Task** | **BP_ND_ Stress Task** | **% Displacement** | **Paired T-test (df=25)** |
| AST | 2.32±0.24 | 2.36±0.27 | (-)1.87±9.87 | t=-0.94 | 2.45±0.49 | 2.30±0.45 | 5.67±9.67 | t=.2.87 |
|  |  |  |  | p=0.36 |  |  |  | **p=0.01** |
| LST | 2.78±0.46 | 2.88±0.38 | (-)6.26±17.14 | t=-1.22 | 2.88±0.66 | 2.71±0.57 | 4.45±17.41 | t=1.66 |
|  |  |  |  | p=0.23 |  |  |  | p=0.11 |
| SMST | 2.45±0.27 | 2.51±0.29 | (-)2.04±9.58 | t=-1.40 | 2.61±0.53 | 2.50±0.47 | 2.96±7.84 | t=2.57 |
|  |  |  |  | p=0.17 |  |  |  | **p=0.02** |
| Whole striatum | 2.42±0.24 | 2.47±0.27 | (-)2.38±9.81 | t=-1.20 | 2.54±0.50 | 2.39±0.43 | 5.04±9.26 | t=2.74 |
|  |  |  |  | p=0.24 |  |  |  | **p=0.01** |

Data are expressed as mean ± standard deviation. AST: associative striatum; LS: limbic striatum; SMST: sensorimotor striatum. There were no significant group differences in BP_ND_ in the control condition.

**Table S4. DA release study, Canada Site: Post-hoc partial correlation between dopamine release ([11C]-(+)-PHNO displacement) in the whole striatum and perceived stress measured by the Trier Inventory for the Assessment of Chronic Stress, with clinical group as covariate (n=54).**

| **Perceived Stress** | **Correlation with dopamine release** |
| --- | --- |
| Work Overload | **r = 0.31, p = 0.03** |
| Social Overload | r = 0.19, p = 0.18 |
| Perception of being overextended | r = 0.20, p = 0.16 |
| Lack of social recognition | r = 0.26, p = 0.07 |
| Work discontent | r = 0.16, p = 0.25 |
| Social Tension | r = 0.18; p = 0.21 |
| Work Pressure | **r = 0.32; p = 0.02** |
| Social Pressure | **r = 0.36; p = 0.01** |
| Social Isolation | **r = 0.38; p = 0.01** |
| Worry Propensity | r = 0.23; p = 0.10 |
| Total score | **r = 0.35; p = 0.01** |

**Montreal Imaging Stress Task (MIST)**

Briefly, subjects performed mental arithmetic on a computer screen that displayed information about the total number of errors, expected average number of errors, time spent on the current problem, and performance feedback for each problem (correct, incorrect, timeout). During the stress condition, subjects completed 6 blocks of 6-minute segments of arithmetic while lying in the scanner. The time constraint was adjusted to be slightly beyond each individual’s abilities, with the average performance set at 20-30% correct answers. In addition, subjects were given negative verbal feedback by the investigator for ~2 min between each block, verbatim language was used in all experiments. Prior to the stress-task, all subjects performed the Sensory Motor Control Task (SMCT) PET session (non-stress), a similar arithmetic task but without time constraints or negative verbal feedback.
